# A novel and cost-effective *ex vivo* orthotopic model for the study of human breast cancer in mouse mammary gland organ culture

**DOI:** 10.1242/bio.051649

**Published:** 2020-05-29

**Authors:** Akash Gupta, Geetanjali Gupta, Rajeshwari R. Mehta, David Z. Ivancic, Rashidra R. Walker, Jankiben R. Patel, Karen M. Gallegos, A. Michael Davidson, Seema A. Khan, Rajendra G. Mehta, Syreeta L. Tilghman

**Affiliations:** 1 Department of Medicine, University of Arizona, Tucson, AZ 85724, USA; 2 Cancer Biology Division, IIT Research Institute, Chicago, IL 60616, USA; 3 Department of Surgery, Feinberg School of Medicine, Northwestern University, Chicago, IL 60611, USA; 4 Division of Basic Pharmaceutical Sciences, College of Pharmacy and Pharmaceutical Sciences, Florida A&M University, Tallahassee, FL 32307, USA

**Keywords:** Breast cancer model, Orthotopic model, Organ culture, Mammary gland

## Abstract

Mouse mammary organ culture (MMOC) is used to evaluate the efficacy of chemopreventive agents against the development of carcinogen-induced preneoplastic lesions and is highly correlative to *in vivo* carcinogenesis models. Here, we developed a new *ex vivo* MMOC model, by introducing human breast cancer cells into the mouse mammary gland. This novel model, termed human breast cancer in MMOC (BCa-MMOC), mimics *in vivo* orthotopic breast cancer mouse models. To develop this model, estradiol- and progesterone-sensitized female mice were injected with letrozole-sensitive and -resistant T47D breast cancer cells in the mammary glands and then euthanized. The glands were cultured *in vitro* with hormone-supplemented media. On day 25, the glands were fixed and processed by histopathology and immunohistochemistry to evaluate for the presence of T47D cells, growth pattern, cancer markers and estradiol responsiveness. Histopathological analyses demonstrated an identical pattern of growth between the breast cancer cells injected *ex vivo* and *in vivo*. Interestingly, clusters of cancer cells in the mammary gland stroma appeared similar to those observed in human breast tumors. The injected T47D cells survived and proliferated for 15 days maintaining expression of estrogen receptor alpha (ER), progesterone receptor (PR), epidermal growth factor receptor (EGFR), and aromatase. The aromatase-overexpressing T47D grown in the BCa-MMOC sufficiently metabolized estrogen, resulting in enhanced cell proliferation, induction of estrogen target genes (i.e. ER and PR-B), and showed typical changes to estrogenic milieu. In summary, here we show a novel, inexpensive *ex vivo* model, to potentially study the effects of therapeutic agents on cancer cells grown in an orthotopic micromilieu.

This article has an associated First Person interview with the first author of the paper.

## Abstract (precis)

**Summary:** Here, we describe and validate a novel, cost-effective *ex vivo* orthotopic model to evaluate chemotherapy agents for breast cancer.

## INTRODUCTION

Mammary glands undergo morphological and biochemical changes during various physiological stages of life, specifically during the transition through nulliparity, pregnancy, lactation and involution (Guzman et al., 1999; Medina, 1996; Nandi et al., 1995). These events are both highly coordinated and regulated; each stage of the transition responds to various hormones (i.e. prolactin, insulin, adrenal corticoids) including ovarian hormones such as estrogen and progesterone. To study mammary gland development, the mouse mammary organ culture (MMOC) model was originally established in mice. In the past, this model proved to be a useful tool to analyze the action of various hormone-mediated inductions of proliferation, lobular alveolar differentiation, secretion and related biochemical events in mouse glands (Mehta et al., 2008). Previous reports demonstrate that the MMOC could be successfully maintained long term in *ex vivo* conditions for at least 30 days without any signs of cellular and structural degeneration (Harbell et al., 1977). Currently, a battery of *in vitro* models or biological assays are used to initially evaluate potential chemopreventive compounds and then select promising anti-cancer agents for development. However, there is an increasing challenge to develop new pre-clinical research models for breast cancer that are accurate, reliable, inexpensive and efficient for the screening of anti-cancer agents. The fundamental criteria for selection of *in vitro* assays includes time and cost effectiveness, controlled test conditions, relevance to organ system and ease of quantitation (Steele et al., 1996) as well as robust clinical correlation. Mehta and colleagues have successfully used the MMOC model to screen various chemopreventive agents for the past two decades and have demonstrated that this model is relevant, reliable and inexpensive (Mehta et al., 2008). Using this model, the chemopreventive efficacy of various chemical or naturally isolated agents were evaluated based on their potential to suppress hyperplastic, mammary ductal or lobular alveolar lesions induced in the presence of various hormonal milieu (aldosterone or estradiol or progesterone) following exposure to chemical carcinogens such as Dimethylbenz(a)anthracene (DMBA) (Mehta et al., 2001). Hyperplastic lesions appeared in the MMOC model after treatment with carcinogens. Additionally, hormonal treatments were comparable to the preneoplastic lesions described by Medina in *in vivo* models, in which prolonged hormonal stimulation of mouse mammary glands led to the development of ductal hyperplasia or hyperplastic alveolar nodules with the later lesions being similar to those induced after carcinogen exposure (Medina, 2000). The hyperplastic lesions developed in the MMOC model were tumorigenic, as they formed adenocarcinomas when transplanted to syngeneic mice (Telang et al., 1979). The efficacy of the chemopreventive drugs observed in the MMOC was highly correlative to *in vivo* screening (Mehta et al., 2008, 2013). Thus, the MMOC model has great translational implications to predict the potential efficacy of promising anti-cancer drugs. Ultimately, selection of such agents could lead to future pre-clinical testing or clinical trials. While the MMOC model has certain drawbacks, such as the inability to explore bioavailability or metabolism of experimental drugs, it is a cost effective and reliable model to pre-screen new chemopreventive agents for breast cancer.

Here, we describe a new model that provides a novel *ex vivo* technique, which can be utilized to study the effects of the tissue microenvironment on proliferation of breast cancer cells and its development inside the mouse mammary gland. To develop this original model, we utilized letrozole-sensitive and -resistant T47D human breast cancer cells, injected them into mouse mammary glands and cultured them *ex vivo*. We characterized and validated this model. Our results confirm that the T47D lesions maintain a comparable phenotype of all evaluated criteria including tumorgenicity, proliferation, estrogen response elements (ERE), transcriptional activity, expression of aromatase, progesterone receptor, epidermal growth factor receptor (EGFR) and hyperplasia.

## RESULTS

### Tumorgenicity and ERE transcriptional activity in T47D derivative cell lines

**Fig. 1.**
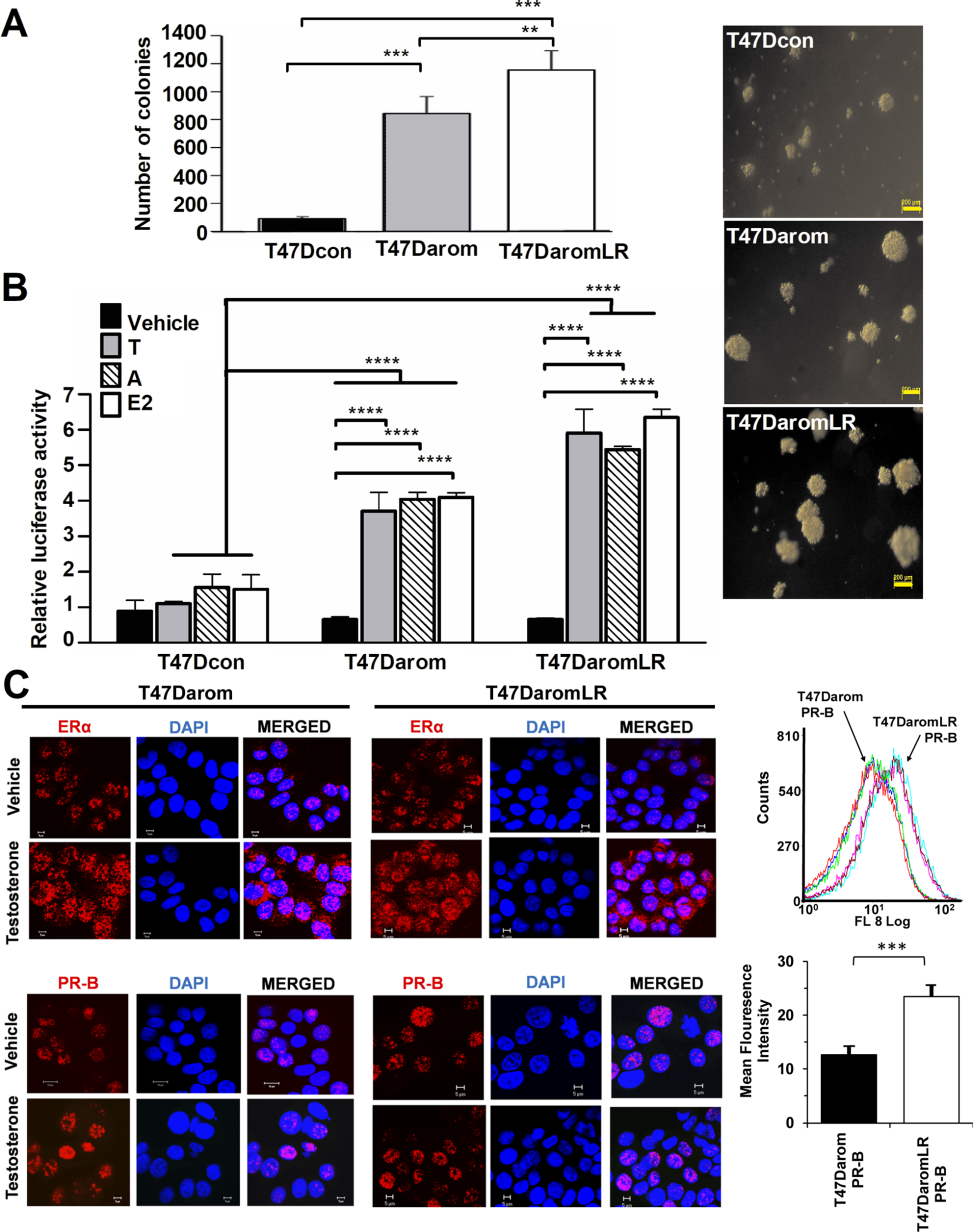
**Effect of hormonal therapy on viability and ERE promoter activity in T47D derivative cell lines.** (A) T47D derivative cell lines were cultured as indicated in the methodology section. Anchorage independent growth was assessed by the colony formation assay. The plot represents the mean±s.d. of the number of colonies of three independent experiments in triplicate. The pictures are representative images of the colonies formed by each T47D cell line. (B) T47D derivative cell lines were transfected with an ERE-luciferase reporter plasmid along with pRL-TK plasmid. After 6 h of transfection, cells were treated with either vehicle, 1 nM testosterone (T), 1 nM androstenedione (A), 1 nM estradiol (E2) in culture medium. After 24 h, dual-luciferase reporter activities were quantified. The firefly luciferase reporter activity was normalized to the Renilla luciferase activity in all the cell lines to account for the variations in transfection efficiencies. Statistical significance differences are shown: ***P<0.001, **P<0.01, *P<0.05. (C) ERα and PR-B expression levels were measured by immunofluorescent staining in both T47Darom and T47DaromLR cell lines in response to testosterone. The cells were plated on glass coverslips, allowed to attach for 24 h, and then treated with 1 nM testosterone for 24 h. Later, the cells were fixed and processed for immunofluorescence staining as detailed in the Materials and Methods. ERα and PR-B are shown in red. Nuclei are counterstained with DAPI in blue. Histograms represent mean fluorescent intensity following staining with anti-PR-B primary antibody and Alexa 594 labeled secondary antibody. Results were quantified and graphed on the right.

To determine tumorgenicity and growth pattern upon testosterone stimulation of T47D derivative cell lines, colony formation assays were initially performed using the T47Dcon, T47Darom and T47DaromLR cells. As shown in Fig. 1A, the T47Dcon colonies that formed were smaller in size and lower in number (<200 colonies) compared to the other cell lines. Only a few colonies observed were ≥200 µm. Compared to the T47Dcon colonies there was a significant increase in number and size of the T47Darom (>800 colonies) and T47DaromLR cells (>1100 colonies) with the T47DaromLR colonies exhibiting the largest size and highest number of colonies.

Previous studies using the T47D variant cell lines prompted the examination of ERE promoter activity in response to aromatase expression in aromatase inhibitor-sensitive cell lines versus aromatase inhibitor-resistant cell lines. As such, the T47D variant cell lines were treated with 1 nM androstenedione (i.e. the substrate for the aromatase enzyme) or 1 nM testosterone and the ERE-luciferase transcriptional activity was measured by luciferase activity. Since T47D cells are estrogen dependent, estradiol was used as a positive control. As demonstrated in Fig. 1B when the T47Dcon cells were stimulated with androstenedione or estradiol there was a two-fold increase in ERE promoter activity, however, testosterone treatment did not cause any significant change in ERE promoter activity. This was likely due to the lack of conversion to estrogen in the T47Dcon cells, which are virtually void of aromatase. When the T47Darom cells were treated with testosterone, androstenedione or estradiol, the relative ERE promoter activity was four-fold higher than the vehicle treated T47Darom cells. Interestingly, when compared to the T47Dcon cells, the T47Darom cells expressed significantly higher changes in ERE transcriptional activity after induction with all of the hormonal treatments. When the T47DaromLR cells were stimulated with testosterone, androstenedione, or estradiol they exhibited more than a five-fold increase in ERE transcriptional activity. This was anticipated since we previously demonstrated that the T47DaromLR cells have a two-fold higher aromatase expression compared to the T47Darom cells (Gupta et al., 2013). Among the T47D variant cell lines the T47DaromLR exhibited the most dramatic increase in ERE transcriptional activity followed by the T47Darom cells. Together, these results indicate that the T47Darom and T47DaromLR are more responsive to testosterone or androstenedione, than T47Dcon cells. This was expected since both the T47Darom and T47DaromLR cell lines are ER+/PR+ and overexpress aromatase, the substrate required for aromatization of androgens to estrogens. After conversion of androgens to estrogens, the estrogen product induces ERE transcriptional activity.

In order to examine the ERα and PR-B expression levels, T47D genetic variants were treated with 1 nM testosterone for 48 h and the hormone receptor status was evaluated by immunofluorescent staining. As illustrated in Fig. 1C, after treatment with testosterone the T47Darom cells showed increased ERα protein expression. Similar results were observed in T47DaromLR cells. The ERα protein expression studies were consistent with our previously published results (Gupta et al., 2013). Since the PR is an ERα responsive gene, upregulation of the ERα protein, in response to testosterone treatment, may translate to increased PR expression. In addition, previous research suggested that an increase in PR-B expression in breast cancer cells, led to enhanced cell proliferation (Kastner et al., 1990; Petz and Nardulli, 2000; Schultz et al., 2003). Therefore, we sought to evaluate whether aromatase levels or letrozole resistance alters PR-B expression after testosterone stimulation. As illustrated in Fig. 1C, T47Darom and T47DaromLR cells have enhanced expression of PR-B (in red) when treated with 1 nM testosterone. These results suggested that in both genetic variants, ERα is functional and responded to higher intracellular estradiol concentration produced by the aromatization of testosterone to estradiol. Overall, PR-B expression was increased in both T47Darom and T47DaromLR cells with higher levels in the T47DaromLR cells. After validating the functionality of the ERα and PR-B, these established cell lines were utilized in the development of the new model for breast cancer drug evaluation using mouse mammary glands.

### Development and characterization of the breast cancer in MMOC (BCa-MMOC) model

**Fig. 2.**
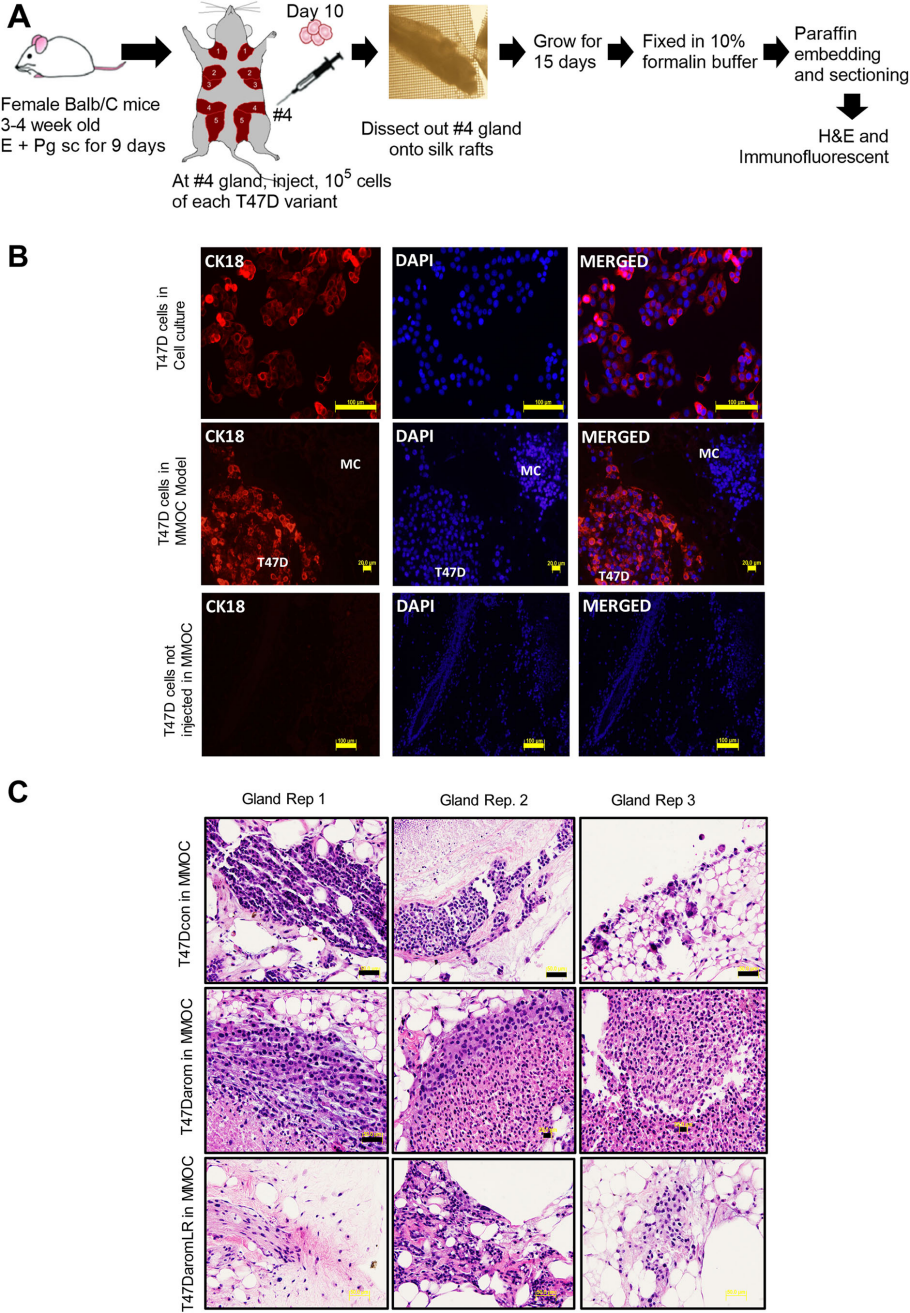
**Scheme of the development and characterization of the BCa-MMOC model.** (A) Schematic of the development of the model where mice were primed by injecting estradiol (E2) and progesterone (Pg) subcutaneously (s.c.), followed by inoculating T47D variant cells into the fourth mammary gland. The mammary gland was removed, cultured and then processed for H&E and immunofluorescent staining. (B) Identification of human breast cancer cells from mouse cells in BCa-MMOC model. CK18 expression used to identify human T47Darom cells in *in vitro* cell culture (top panel), *ex vivo* (BCa-MMOC) (middle panel), and *ex vivo* (BCa-MMOC) negative control (bottom panel), in the absence of T47Darom cell injection into the mouse mammary gland. Cells were plated on glass coverslips, then fixed with formalin and processed for immunofluorescence staining of CK18 in red. Nuclei were counterstained with DAPI in blue. For the *ex vivo* immunofluorescent staining the number 4 gland of BALB/c mice were injected with T47Darom cells and grown using the BCa-MMOC condition for 15 days. (C) T47Dcon, T47Darom or T47DaromLR cells were injected in the number 4 mammary glands and H&E staining was performed. Panel shows three different repeats of mammary glands for each cell line. All images were taken with a 20× objective lens using an Olympus BX40 microscope, where HC represents human cells and MC represents mouse cells.

For these experiments, the T47D genetic variant cell lines (T47Dcon, letrozole-sensitive T47Darom and letrozole-resistant T47DaromLR) were injected into the fourth pair of mammary glands of BALB/c mice. They were then cultured *ex vivo* for 15 days in the presence of various hormones, as described in the Materials and Methods section. Fig. 2A summarizes the experimental design employed to develop the BCa-MMOC system. To evaluate the presence of the human breast cancer cells in the BCa-MMOC, it was necessary to distinguish between human cells and mouse cells. Therefore, a CK18 monoclonal antibody which detects the human epithelial cell marker, cytokeratin 18 (CK18) was initially used. To accomplish this, the T47Darom cells were grown on a cover slip, then fixed and stained for the expression of the human specific CK18 protein by immunofluorescence. As shown in the upper panel of Fig. 2B, the T47D cells show distinct cell surface expression of CK18 suggesting that the T47D cells are positive for CK18 expression (shown in red), confirming this as a suitable biomarker to identify and distinguish human breast cancer cells from mouse mammary gland cells. The nuclei were also counterstained blue with DAPI. After confirming CK18 expression in T47D cells, the number 4 glands of the BALB/c mice were injected with all three cell lines and cultured for 15 days. The whole glands were excised, fixed and embedded into paraffin blocks for immunohistochemical detection. These studies were designed to distinguish the T47D breast cancer cells of human origin from mouse mammary gland cells as well as identify the pattern of breast cancer cell distribution and growth. Next, to further confirm that CK18 is a human specific marker, whole mouse mammary glands lacking T47D cells were stained for CK18, and results demonstrated that the glands did not stain positive for CK18 (Fig. 2B). Immunofluorescent staining was only present when T47D cells were injected into the MMOC suggesting that CK18 is a suitable and reliable biomarker for tracking human breast cancer cells of luminal origin when injected into mouse mammary glands.

To validate the use of this novel *ex vivo* orthotropic model for breast cancer it was critical to show that once injected, the human breast cancer cells could be cultured *ex vivo*, survive, proliferate and be reproduced in different cell lines. As such, the T47Dcon, T47Darom or T47DaromLR cells were injected in the number 4 mammary glands and Hematoxylin and Eosin (H&E) staining was performed. The three cell lines established well and created a solid tumor-like pattern within the MMOC model (Fig. 2C). Based on the H&E staining images, the overall morphological features are consistent with breast adenocarcinoma. The T47Dcon and T47Darom lesions are well-defined with uniform borders, while the T47DaromLR lesions appear more diffuse and invasive. This difference may be due to the more aggressive nature of letrozole-resistant cells, as previously described (Tilghman et al., 2013). Additionally, this phenomenon was reproduced using triple negative MDA-MB 231 breast cancer cells and compared to normal human immortalized MCF-12F breast cells (Fig. S1).

### Comparison of the BCa-MMOC model with xenografts and breast cancer tissue sections

**Fig. 3.**
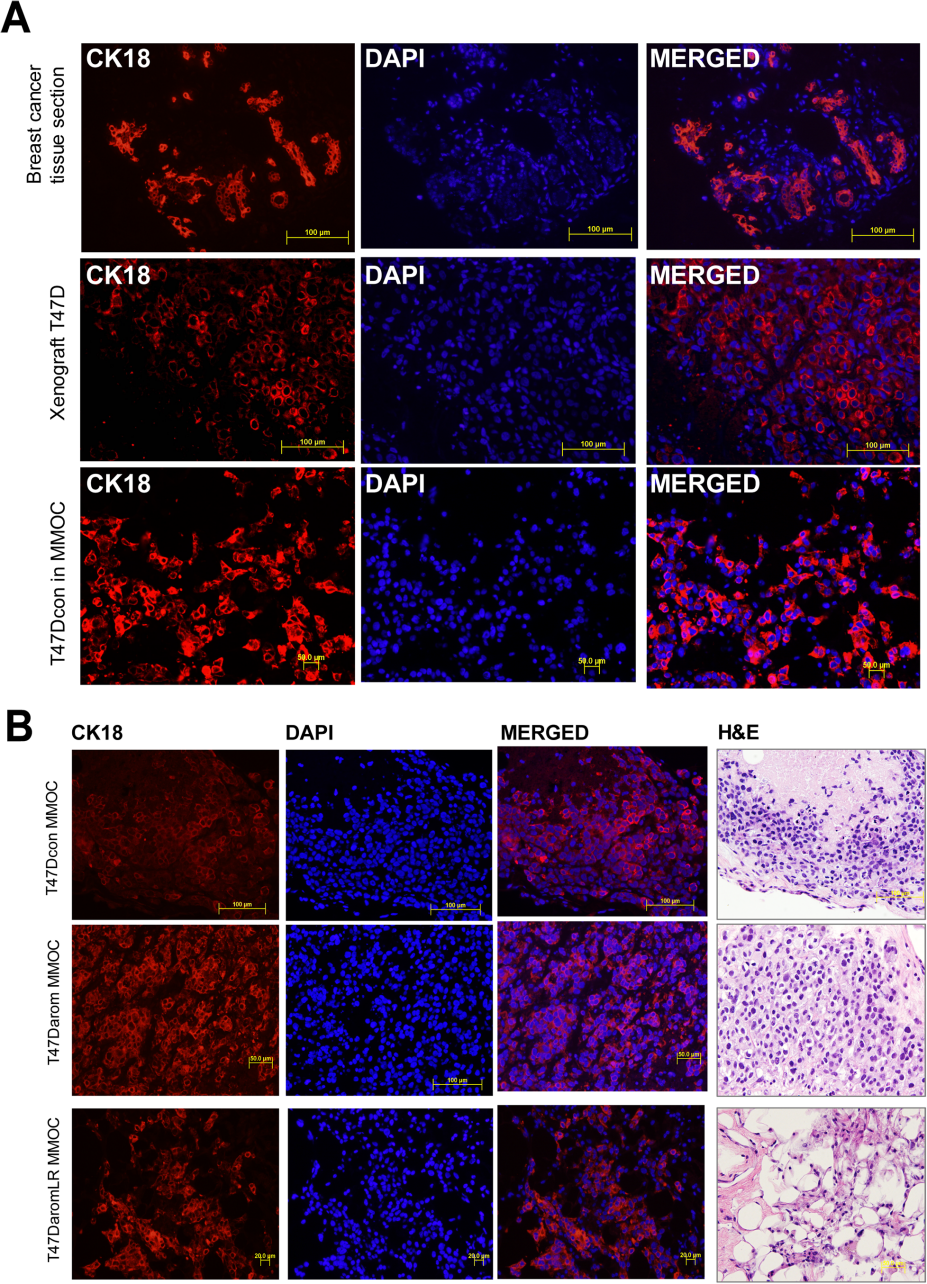
**Comparison of CK18 expression in human breast tumors, human breast cancer xenografts and T47Darom cells cultured in BCa-MMOC.** (A) CK18 expression was evaluated in a human breast cancer patient specimen, xenograft of T47D cells in nude athymic mice and mouse mammary glands injected with T47D cells grown in BCa-MMOC. Paraffin sections of respective tissues were processed for CK18 immunofluorescence staining where the left panel represents CK18 (in red) expression, the middle panel represents DAPI (in blue) nuclear expression and the right panel is the merged images of CK18 and DAPI expression. (B) T47Dcon, T47Darom and T47DaromLR cells used in the BCa-MMOC model and processed for CK18 and DAPI immunostaining. The figure is representative of one of the three repeats of the mouse mammary glands for each cell line.

Next, it was equally important to verify that the cancer cells in the BCa-MMOC model retained their original characteristics. To do so, it was necessary to compare this new model to other well established and widely used models such as xenografts in mice and human breast cancer tissue sections. To this end, the distribution and growth pattern of the T47D cells grown in the BCa-MMOC were compared to either T47D xenografts in nude mice or histopathological sections of human breast tumor tissue (Fig. 3A). To confirm human origin, all of the samples were stained for CK18 protein expression by immunofluorescence. There was intense CK18 staining in the human breast cancer tumor tissue of lobular origin as well as the T47D breast tumor tissue in athymic mice. Interestingly, the T47D cells cultured in the BCa-MMOC exhibited a similar pattern of expression and distribution as to that of the T47D cells in the xenograft model. These results indicated that the T47D cells grown within the BCa-MMOC model, follow similar growth patterns to those seen in the xenograft model as well as human breast tumor tissue. In order to detect the uptake of the T47Dcon, T47Darom and T47DaromLR cells within the number 4 mammary gland, their growth pattern was assessed by H&E and CK18 immunofluorescent staining (Fig. 3B). All of the T47D variant cell lines were positively stained for CK18 confirming the BCa-MMOC exhibited a suitable and permissive environment for the growth of the T47D cells.

**Fig. 4.**
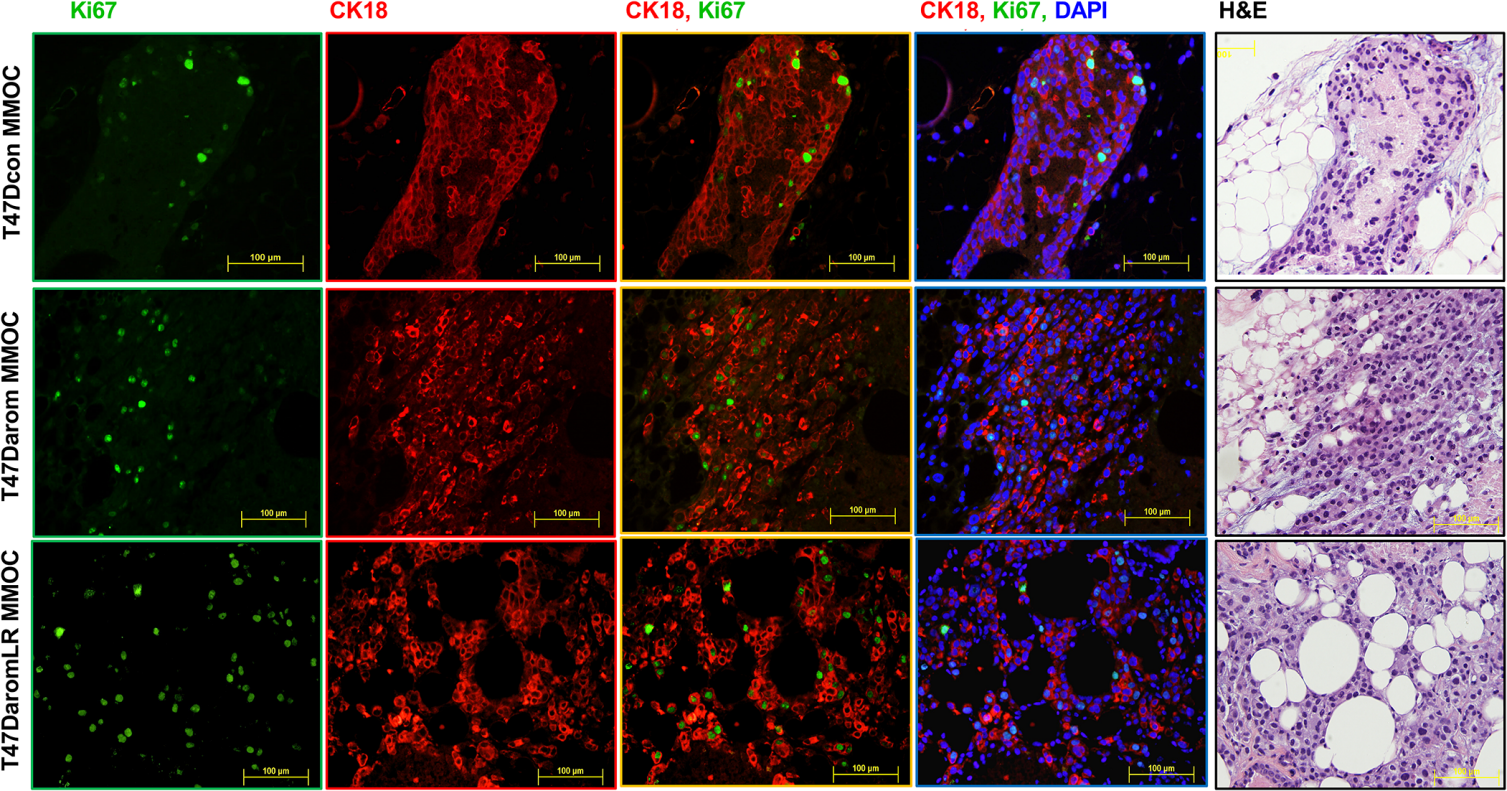
**Survival and proliferation of T47Darom cancer cells in Ki67 positive cells within the BCa-MMOC model.** T47Dcon, T47Darom and T47DaromLR cells were injected in the BCa-MMOC model and evaluated by Ki67 staining (in green), CK18 staining (in red), H&E staining, and DAPI counterstaining (in blue). All images were taken using a 20× objective lens.

To evaluate cell proliferation and survival status within the mouse mammary gland organ culture, Ki67 was used as a biomarker. As such, the T47D variant cell lines were grown in the BCa-MMOC and stained for Ki67 and CK18. As illustrated in Fig. 4, the cells showed positive CK18 (red) expression on the cell surface while the nuclei were stained with DAPI (blue). The cells were dually stained with Ki67 (green) and CK18 and strongly expressed both proteins. This result suggests that the T47D variant human breast cancer cell lines retain proliferative competence when injected and cultured within the mouse mammary gland. Overall, this result confirms that all three T47D variant cells lines cultured in the mammary gland for 15 days continue to survive and proliferate.

### Aromatase, progesterone receptor, and EGFR expression in T47D variants cultured in BCa-MMOC model

**Fig. 5.**
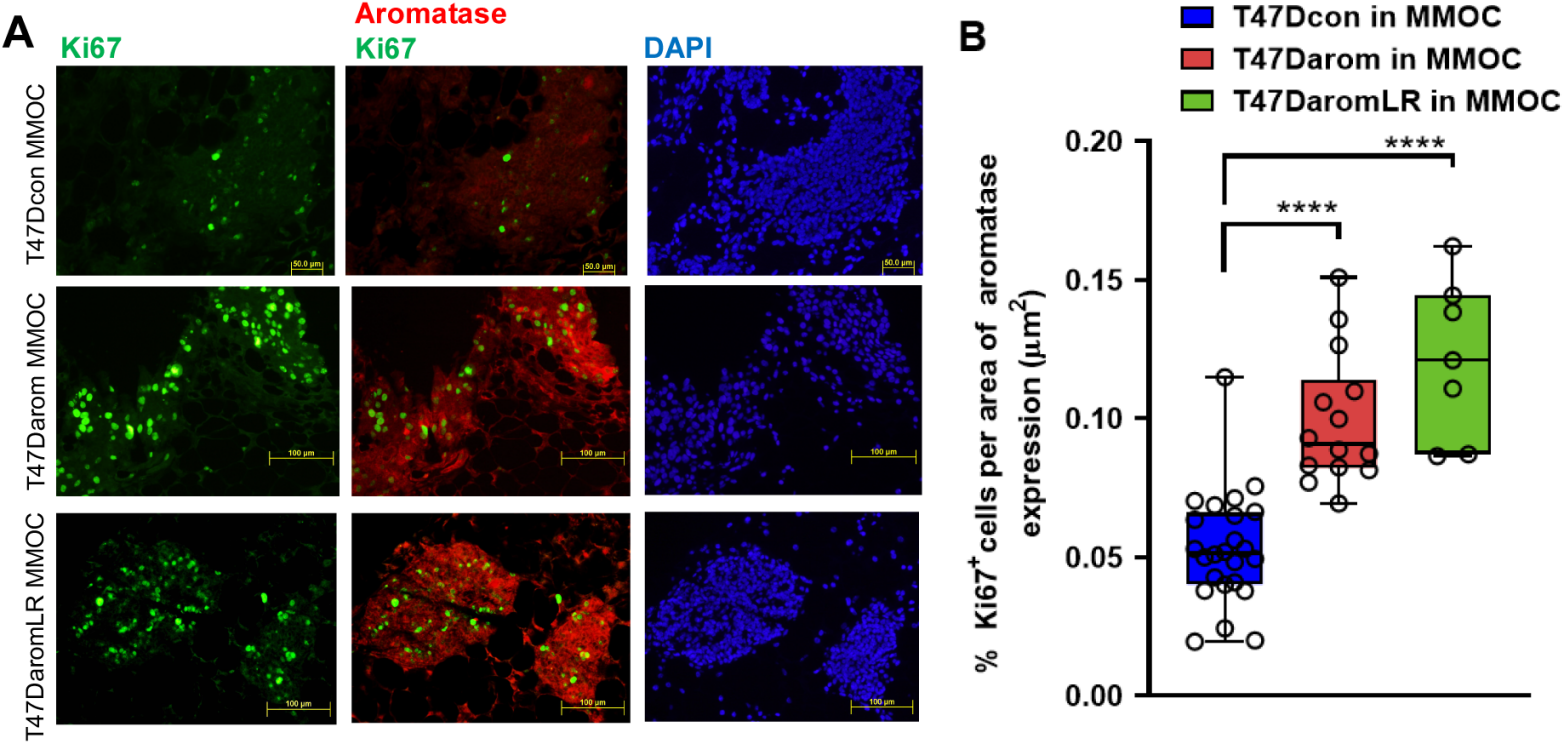
**Immunofluorescence analysis of aromatase and Ki67 in BCa-MMOC section.** (A) T47Dcon (top panel), T47Darom (middle panel) and T47DaromLR (bottom panel) were grown in BCa-MMOC and mammary gland sections and representative images were dually immunostained for Ki67 (in green) and aromatase (in red). All images were taken at 10× objective. (B) Quantitative analysis of the area of aromatase expression occupied by T47Dcon, T47Darom and T47DaromLR expressing aromatase (in red) within the mouse mammary gland. Results were quantitated and expressed as the percentage of proliferation (as measured by Ki67 positive cells µm^−2^ area occupied by the T47D variant cells expressing aromatase, ****P<0.0001). Five glands for each cell type were measured for aromatase and Ki67 staining.

Since the T47Darom cells were introduced in the mouse mammary gland, it was necessary to assess and confirm whether these cells expressed functional aromatase protein and determine if their proliferation was similar to the T47Darom cells cultured *in vitro* (Fig. 1A; Fig. S2). Endogenous aromatase expression was measured in the T47D variant cell lines (Fig. S3) and since the variants express differential levels of aromatase, they were injected into the BCa-MMOC model and dual immunofluorescent staining of these sections was performed. Both aromatase overexpressing cells lines (T47Darom and T47DaromLR cells) were intensely stained for aromatase (in red) and nuclear Ki67 (in green) compared to T47Dcon cells (Fig. 5A). The percentage of Ki67 positive cells µm^−2^ area of the aromatase expressing T47D variant cells was quantified (Fig. 5B). Box and whisker plots indicate a higher percentage of Ki67 positive cells in the T47Darom and T47DaromLR cell lines compared to the T47Dcon cells. It is possible that aromatase expression enhances cell proliferation due to the conversion of androgens to estrogen in the cells via aromatase. However, it is important to note that the T47Dcon cells exhibited decreased Ki67 nuclear staining with negligible to no detectable aromatase expression (top right panel). In general, T47Dcon cells had a lower number of cells with Ki67 expression compared to T47Darom cells as well as a lower proliferation index as observed *in vitro*, which was confirmed by previous research (Gupta et al., 2013).

In order to ascertain the functionality of the BCa-MMOC model, proof of concept studies were conducted to investigate whether estrogen receptor signaling remained intact after the cells were injected and cultured *ex vivo* for 15 days. To this end, the T47D variant cell lines were stained for PR-B. As demonstrated in Fig. S4, all of the variant cell lines injected into the mammary glands were positive for PR-B expression. This is an important observation as it suggests that ER signaling remained intact as the cells were cultured in an *ex vivo* environment in the mouse mammary glands and retained a similar expression pattern to those observed *in vitro*. Along those lines, a similar result regarding EGFR expression was also demonstrated in Fig. S5.

### Comparison of hyperplasia after DMBA treatment in MMOC culture and in current BCa-MMOC model

**Fig. 6.**
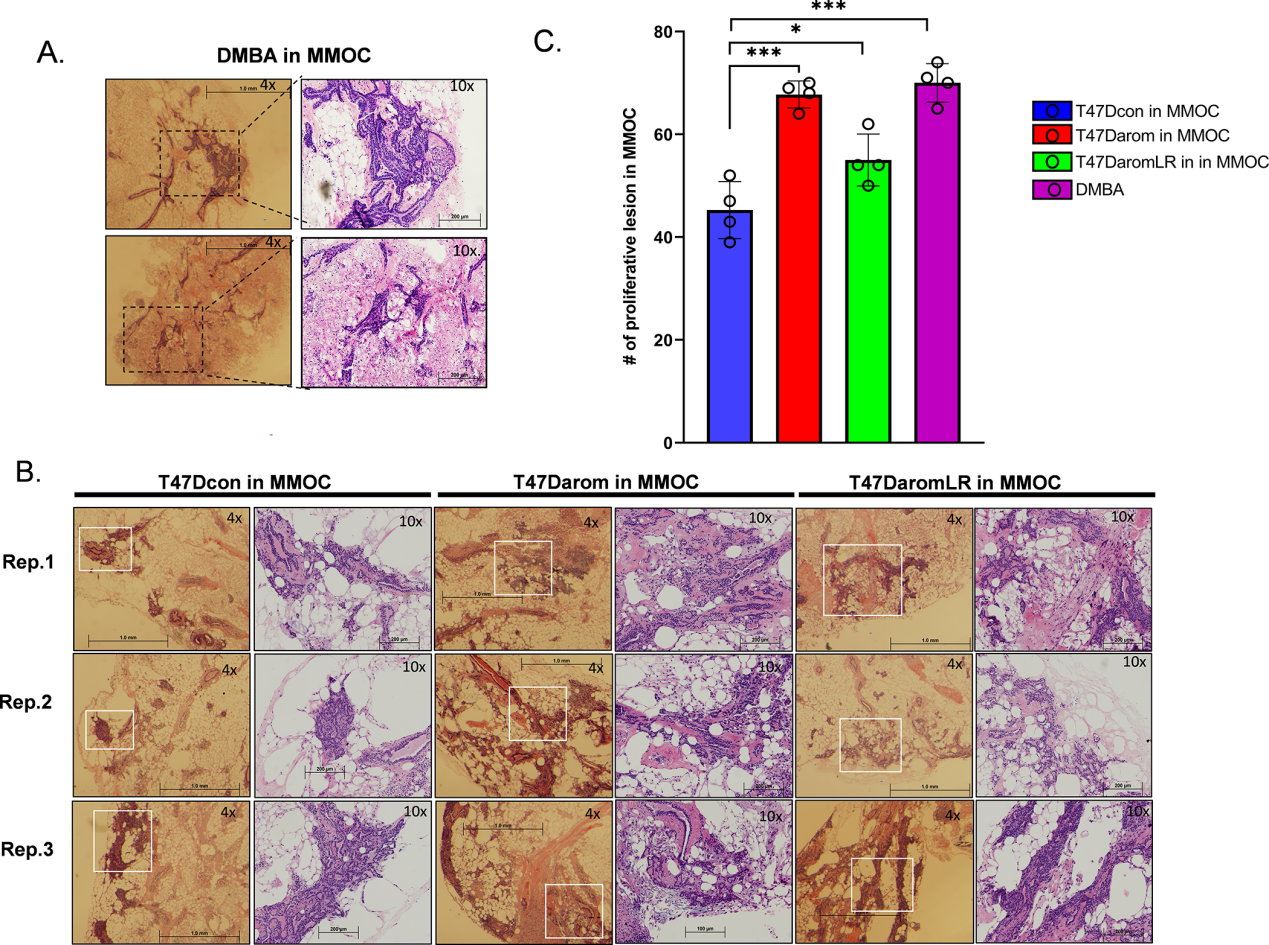
**Histomorphological changes of mouse mammary gland in BCa-MMOC model and its comparison with DMBA treated mouse mammary gland.** (A) The whole mouse mammary gland was grown as BCa-MMOC model with the T47D variant cell lines and was fixed and paraffin embedded. The images were taken at 4× magnification. Similarly, the mouse mammary glands treated with DMBA (5 µg ml^−1^) for 24 h cultured under similar conditions were processed in a similar way to the BCa-MMOC glands. H&E staining was performed in longitudinal sections of the whole glands (bottom panel). Morphological differences in various ductal and lobular growth patterns were observed at low power (4×) using a dissection microscope. The inset area corresponds to the area observed under a 20× objective using a light microscope. (B) Three representative images of H&E staining of the mouse mammary injected with T47D derived cells appear similar to DMBA proliferative lesion taken at 4X power shows lower number of proliferative lesions for T47Dcon (left panels) compared to T47Darom (middle panels) and T47DaromLR (right panels). H&E images of three replicate (rep.) mouse mammary glands in BCa-MMOC are shown for each injected cell lines. (C) Using 4× magnification, the proliferative lesions were counted after the variant cell lines were injected into the mouse mammary gland cultured *ex vivo*. The total number of focal lesions was quantitated for a least three glands per cell lines. Box and whisker plots were generated to express the number of proliferative lesions per T47D variant cell line.

The mouse mammary organ culture and various animal models showed two distinct biological phenotypes of pre-malignant lesions that arise as a result of immortalization and epithelial hyperplasia. These two lesion types are ductal hyperplasia and alveolar hyperplastic nodules. They are distinguished by their pattern of epithelial cell proliferation and their extent of aneuploidy. Induction of these lesions could be due to viral, chemical and hormonal agents. Determination of whether these lesions in animal models mimic human pre-malignant progression was critical. In the present study, it was important to determine the extent and occurrence of these lesions within the mouse mammary gland after exposure to the T47D derivative cells in BCa-MMOC. The glands were exposed to the tumor-promoting agent, DMBA and this treatment was used as a positive control. For these purposes, four sets of the fourth mouse mammary glands were observed for the development of hyperplasia after treatment with DMBA. Afterwards, their size and frequencies were recorded. Fig. 6A shows the whole gland treated with DMBA and cultured in the MMOC conditions. There were more than 60 hyperplastic lesions identified with a size >1 mm. As expected, the frequency of the DMBA induced lesions was higher in number than the vehicle treated samples. In general, compared to the vehicle treated mouse mammary glands, these lesions are considered pre-neoplastic, since they fail to regress after the removal of growth promoting hormones. In the present study, observations were made when the T47Dcon, T47Darom and T47DaromLR cells were injected in the mouse mammary glands (Fig. 6B). The presence of T47Dcon cells in mouse mammary glands lead to the development of hyperplastic lesions originating from the mouse cells. The size of the lesions ranged from 0.2 mm to 0.8 mm. Compared to the T47Darom and T47DaromLR cells, the T47Dcon cells had a lower number of lesions. In comparison, the mammary glands injected with T47Darom and T47DaromLR cells showed the formation of hyperplastic lesions >1 mm in size, like those observed in DMBA-treated mammary glands. In addition, the frequency of larger hyperplastic lesions originating from the mouse cells, were more prominent in the mammary glands injected with T47Darom and T47DaromLR cells, when compared to T47Dcon cells. These results suggest that the presence of T47Darom and T47DaromLR cells enhance hyperplasia of mouse mammary glands more efficiently than T47Dcon cells. It is evident that both T47Darom and T47DaromLR cells may enhance epithelial cell proliferation of the ducts and alveoli in the host gland with the former leading to the formation of hyperplasia, appearing like ductal carcinoma *in situ*. These observed lesions are not prominent in glands injected with T47Dcon cells. However, the cause of increased hyperplastic lesions in mouse mammary glands injected with T47D derivative cells remains undetermined.

## DISCUSSION

Previous studies of *in vivo* orthotopic models, using human breast cancer cells transplanted in the mouse mammary gland fat pad, were primarily developed to observe the interaction between mammary gland epithelium and the surrounding stroma using tissue recombination techniques (Sakakura, 1987). Histologically, the mouse mammary stroma is dramatically different from human stroma. In the mouse, the stromal tissue is composed mostly of adipose tissue, whereas in humans it is mainly fibroblastic (Topper and Freeman, 1980). Hence, it is difficult to study the stromal-epithelial interaction in transplanted models (Jensen and Wellings, 1976).

Several other models were developed using the mammary fat pad of athymic mice (Parmar and Cunha, 2004). In one study, human benign breast lesions were transplanted in the mammary fat pad of athymic mice in order to observe the growth pattern of the ductal epithelium within the xenograft in the presence of hormones, such as estradiol and progesterone. This study showed that the ductal epithelium maintained its normal differentiation and was able to proliferate in the presence of hormones (McManus and Welsch, 1984). However, attempts to simulate a similar model to observe the developmental pattern of human ductal organoids were disappointing (Sheffield and Welsch, 1988).

In a separate study, Weinberg's group described the orthotopic xenograft model in which both stromal and epithelial components of human origin were reconstructed in mouse mammary glands (Kuperwasser et al., 2004). The idea was to genetically generate the human stromal component, in order to study its effect on normal human breast epithelial cells. In this study, the fibroblasts were immortalized by overexpressing telomerase. Additionally, growth factors were overexpressed, followed by mammary fat pad injections. When normal human breast epithelial organoids were injected in the humanized fat pad, they proliferated and developed structures similar to malignant lesions in the human breast. On the other hand, the fibroblasts injected without overexpression of growth factors failed to support the growth of premalignant/malignant-like lesions from epithelial cells. The humanized mouse mammary model described by Weinberg's group, demonstrated the influence of stromal tissue microenvironment in the development and progression of normal breast cells into a more invasive phenotype (Kuperwasser et al., 2004). Even though this humanized model has significant importance, it is an *in vivo* model that requires expertise to generate the appropriate stromal components in mice and is further limited by time (2–3 months) and cost.

In this study, we now generate an original *ex vivo* orthotopic model, using the MMOC that was designed to study the growth pattern of aromatase overexpressing and aromatase inhibitor resistant cells. This model provides conditions similar to those described *in vivo* and has been characterized for the identification of mouse luminal epithelial cells. We have shown here that the cells injected into the mammary fat pad are maintained in the MMOC as indicated by their continued proliferation. Additionally, the injected breast cancer cells maintained their original characteristics such as sustained expression of ER (i.e. as demonstrated by positive expression of PR-B), EGFR and overexpression of aromatase. Enhanced expression of EGFR during the acquisition of resistance towards aromatase inhibitor has been previously reported (Johnson et al., 2013). Since the presence of EGFR was previously confirmed in T47DaromLR cells *in vitro* (Gupta et al., 2013), it was essential to verify whether the T47DaromLR cells displayed a similar pattern of EGFR expression when grown in the BCa-MMOC model. As such, EGFR expression was maintained in the T47DaromLR cells when cultured in the *ex vivo.* The *ex vivo* setting provides additional confirmation that the original characteristics of the cells cultured *in vitro* are maintained *ex vivo*. Further, when both the T47Darom and T47DaromLR cells were cultured *in vitro* they exhibited an increased proliferative index (as demonstrated by an increase in the number and size of the foci) compared to the T47Dcon cells. Similar observations were observed in the T47Darom and T47DaromLR cells cultured in the BCa-MMOC model, in that both cell lines exhibited increased nuclear Ki67 staining. The Ki67 staining was not only indicative of cells proliferating, but also the BCa-MMOC model provided a permissive environment for growth of the T47D derivative cell lines. Although letrozole sensitivity and resistance of the T47D variant cells was not directly measured in the BCa-MMOC model, the cells cultured *ex vivo* maintained the original phenotypic characteristics as those *in vitro*, suggesting they may retain their original response to letrozole and therefore may be a suitable model for drug screening.

Aromatase overexpression induced morphological changes within the mammary glands, such as hyperproliferation of the ductal and lobuloalveolar region. Similar observations were reported by Tekmal and his group, where mouse mammary glands from aromatase overexpressing transgenic mice showed hyperplastic and malignant breast tumor-like lesions (Diaz-Cruz et al., 2011; Tekmal et al., 1999). In addition, when the T47D variant cell lines where cultured in the BCa-MMOC we observed growth patterns that were similar to other orthotopic models of human breast cancer cells transplanted *in vivo* and to that of actual human breast cancer tumors. We validated that the T47D variant cells were capable of being sustained within the BCa-MMOC model by using the CK18 human luminal epithelial marker. CK18 expression positively distinguished the human cells from those of the mouse cells.

Additional characterization of the BCa-MMOC model was performed by comparing the histomorphological characteristics that result from inducing carcinogenesis with DMBA versus injecting the mouse mammary glands with the T47D variant cell lines. The mouse mammary glands were examined and the results from the two approaches were similar; both strategies revealed increased hyperproliferation in the ductal and lobular-alveolar regions. Interestingly, the mouse mammary glands injected with T47Dcon cells show very few hyperplasic lesions. These results suggest that T47Darom and T47DaromLR cells modified the mouse mammary gland stromal environment similar to that observed following tumor-inducer DMBA treatment. Our results validate this novel and efficient method for studying breast cancer. This original model recapitulates many major histological features and many cancer molecular markers found in aggressive breast cancers were retained.

Finally, the BCa-MMOC model is a cost-effective system that can be used to evaluate the aggressive phenotype of breast cancer cells, their ability to affect the microenvironment of the mouse mammary gland, and the formation of dysplastic lesions. Compared to the traditional xenograft models, the BCa-MMOC is significantly more economical. The acquisition and care of the BALB/C mice costs less than that of nude mice, especially since mice are euthanized after 10 days thereby eliminating the need for extended housing, feeding and animal monitoring. This is in stark contrast to xenograft models in which tumors typically form 2–3 months after injection. Finally, the total amount of drug needed for screening is reduced because only the mammary gland is used as opposed to the entire animal. While there are several advantages of the model, it is limited by a few factors including the inability to test the pharmacokinetic properties of compounds. The BCa-MMOC model can only be used to assess the drug in the local mammary gland environment and as such restricts the ability to measure drug efficacy, bioavailability or route of administration. Despite these limitations, the use of this model as a preliminary screening tool outweighs the drawbacks. The data generated from this model could be extrapolated and used to understand drug mechanism of action and/or aid in therapeutic decision-making. Overall, this model is less costly than performing pre-clinical studies in xenograft mouse models. In addition, this model could potentially benefit pharmacological breast cancer research and drug discovery by evaluating the efficacy of novel therapeutics. In conclusion, this study describes the development of a new and inexpensive research tool.

## MATERIALS AND METHODS

### Hormones and stock solutions

Testosterone, androstenedione and estradiol were obtained from Sigma Chemicals (St Louis, MO, USA). The stock solution of 10 mM testosterone, androstenedione and estradiol were prepared in ethanol and stored at −80°C. The working solutions were prepared to give final concentrations of 1 µM and 100 nM for testosterone whereas all other agents working solutions were prepared as 10 mM and stored at −20°C. G418 was purchased from Research Products International Corp. (Palos Heights, IL, USA), dissolved in Phosphate Buffer Saline (PBS) at 100 mg ml^−1^ and then stored at −20°C. The ﬁnal concentration of ethanol in the cell growth medium was <0.01% (v/v), which was conﬁrmed to have no statistically signiﬁcant effect on cell growth.

### Cell lines

In this study, we utilized three cell lines previously developed and derived from the T47D parental breast cancer cell line (Gupta et al., 2013). These variant cell lines include the T47Dcon (T47D cells stably transfected with empty pcDNA3.1 plasmid with a basal level of aromatase expression), T47Darom (aromatase overexpressing T47D cells) and T47DaromLR (aromatase overexpressing T47D cells that are resistant to the aromatase inhibitor, letrozole). All T47D variant cell lines were cultured at 37°C and 5% CO_2_ in Phenol Red-free minimum essential medium (MEM) (Invitrogen, Carlsbad, CA, USA) supplemented with 10% heat-inactivated fetal bovine serum (FBS) (Atlanta Biologicals, Lawrenceville, GA, USA), 0.01% non-essential amino acids, antibiotics (Invitrogen, Carlsbad, CA, USA) and 600 µg G418 (Research Products International Corp., Palos Heights, IL, USA). The medium was replenished every 48 h and the cells were sub-cultured weekly. Mycoplasma testing has been performed on all cell lines. The T47D variant cells were authenticated by short tandem repeat profiling from ATCC and results verified they shared greater than 85% homology with the T47D cell line. Cell lines with ≥80% match are considered to be related (i.e. derived from a common ancestry). Briefly, 17 short tandem repeat (STR) loci plus the gender-determining locus, Amelogenin, were amplified using the commercially available PowerPlex® 18D Kit (Promega). Samples were processed using the ABI Prism® 3500 xL Genetic Analyzer and analyzed using GeneMapper^®^ ID-X v1.2 software (Applied Biosystems). Appropriate positive and negative controls were run and confirmed for each sample submitted. Cell lines were authenticated using STR analysis as described in 2012 in ANSI Standard (ASN-0002) by the ATCC Standards Development Organization (SDO) and as previously described (Capes-Davis et al., 2013)*.*

### Immunofluorescence staining and confocal microscopy

For immunofluorescent detection of various proteins, the T47D variant cells lines were cultured on glass coverslips, fixed in 4% paraformaldehyde for 10 min, washed with PBS and permeabilized in ice-cold 100% methanol for 3 min. To block non-specific binding, cells were incubated with 1% bovine serum albumin (BSA) in PBS for 30 min followed by 1 h incubation with primary antibodies against aromatase H4 (AbD Serotech, Kidlington, UK), estrogen receptor α (ERα) F-10 (Santa Cruz Biotechnology, Dallas, TX, USA), progesterone receptor-B (PR-B) B-30 (Santa Cruz Biotechnology), Epidermal Growth Factor Receptor (EGFR) (Ab-10, clone 111.6) Neomarkers, Freemont, CA), and Ki67 (8D5) (Cell Signaling Technology, CA, USA). All antibodies were diluted per the manufacturer's instructions in PBS containing 0.1% BSA. After incubation with the primary antibody, cells were rinsed extensively with PBS, and then treated with the appropriate secondary antibody at a 1:300 dilution (Invitrogen, Carlsbad, CA, USA). The coverslips containing the cells were washed with PBS and mounted on slides in media containing 4′,6-Diamidino-2-Phenylindole (DAPI, Vector Laboratories Inc., Burlingame, CA, USA). The slides were imaged using an Olympus BX 40 microscope (Olympus, North Ryde, Australia) fitted with appropriate filters to detect both Alexa 594 and Alexa 488 and DAPI fluorescence simultaneously and each of the two fluorochromes separately.

For ERα, PR-B and aromatase expression, fluorescent images were obtained using a Carl Zeiss LSM 510 (Thornwood, NY, USA) laser scanning confocal microscope equipped with a 63× water immersion objective. The beams of 364 nm, 488 nm and 568 nm laser were used for excitation, and blue, green and red fluorescence, respectively, and were recorded through LP505, and LP470 filters.

### Quantification of Ki67 positive cells within aromatase expressing cell line

Formalin-embedded mouse mammary gland tissues from each animal injected with T47D variant cells were cut in paraffin sections (5 μm thick) and mounted onto slides. The assessment of human specific aromatase protein (red) and Ki67 proliferation protein (green) was conducted using dual immunofluorescence staining for each BCa-MMOC gland section. The AxioVision LE software on Zeiss Brightfield microscope system was used to measure area of aromatase expressing cells in µm^−2^ using six different images from different fields taken at 10X objective for T47Dcon, T47Darom, and T47DaromLR within MMOC. Afterwards, the Ki67 positive cells (in green) were counted within the area of aromatase expressing cells and the percentage was derived for each cell line. Results were quantitated and expressed as the percentage of proliferation (as measured by Ki67 positive cells/µm^2^ area occupied by the T47D variant cells expressing aromatase, ****P<0.0001). Five glands for each cell type were measured for aromatase and Ki67 staining.

### Colony formation assay

The growth pattern of the three T47D variant cell lines (T47Dcon, T47Darom and T47DaromLR) were validated by performing the soft agar assay, according to the Katz et al, modified procedure (Katz et al., 2008). Anchorage-independent growth was determined for the cells treated with 1 nM testosterone in a six-well plate. A mixture of 0.5 ml of pre-warmed (37°C) Phenol Red-free 2× MEM supplemented with 20% FBS and 0.5 ml pre-warmed (56°C) 1.0% Bacto agar select (0.5% final agar) was added to a 100 µl cell suspension (5000 cells/well) and seeded over a 0.6% agar/ Phenol Red-free 2× MEM supplemented with charcoal-stripped FBS at 20% pre-layer (1 ml) in a six-well plate. Semisolid 0.6% feeder layers (0.5 ml) were overlaid on top of the solidified cell layers. Feeder medium was replaced every 48 h with fresh medium containing 1 nM testosterone. The cells were allowed to grow in a humidified incubator at 37°C with 5% CO_2_ for 21–28 days. The colony numbers were determined for each T47D derivative cell line by measuring the individual colony size. Colonies that were larger than 200 µm were calculated for T47D cell types. The data were collected from pictures of colonies taken in three different fields by using an Olympus SZX7/Olympus ACH 1x microscope with a 4× objective. Each treatment was plated in triplicates.

### ERE-transcriptional activation assay

The dual luciferase assay was conducted as previously described (Qi et al., 2004). Briefly, T47Dcon, T47Darom and T47DaromLR cells (120,000 cells/well) were plated on 24-well plates. After a 24 h incubation period, the cells were transfected with 0.8 μg of ERE-luc and 10 ng of the internal control phRL-TK using Lipofectamine 2000 (Invitrogen) according to the manufacturer's instructions. After 24 h, cells were treated with the vehicle, 1 nM testosterone, 1 nM androstenedione or 1 nM estradiol for a 24 h period, then processed for the firefly and Renilla luciferase activity using the Dual-Luciferase Reporter Assay Kit (Promega, Madison, WI, USA). Luciferase activity was measured by Veritas™ Microplate Luminometer (Turner BioSystems, CA, USA). The relative luciferase activity was expressed as the ratio of the firefly luciferase/Renilla luciferase/mg cell protein.

### Validation and development of BCa-MMOC model

The BCa-MMOC model, where human breast cancer cells are cultured in normal mouse mammary glands in organ culture, has not previously been reported. In this model, the survival and proliferative capacity of the T47D variant human breast cancer epithelial cells (T47Dcon, T47Darom and T47DaromLR) in mouse mammary organ culture were evaluated. To prime the animals for the organ culture assay, 3−4 week-old BALB/c female mice obtained from Charles River Laboratories (Wilmington, MA, USA) were pretreated with 1 µg of estradiol and 1 mg of progesterone for 9 days. This treatment sensitized the mammary glands to respond to the hormones present in the culture medium. On the tenth day, the mice were euthanized and the fourth pair of abdominal mammary glands was injected with 1×10^5^ T47D control, T47Darom or T47DaromLR cells. The glands were dissected onto silk and transferred to 60 mm culture dishes as described previously (Mehta et al., 2008). Each dish contained 5 ml of 10% charcoal stripped-FBS/MEM supplemented with1 μg ml^−1^ progesterone, growth promoting hormones (5 µg insulin, 5 μg prolactin per ml culture medium) and both aseptically dissected fourth abdominal mammary glands on silk raft, which were floated on the culture medium. Based on culture conditions previously described by Harbell et al. (1977) and Telang and Sarkar (1983). The glands were incubated for 15 days at 37°C, 95% O_2_/5% CO_2_ with medium changed on alternate days. After 15 days of incubation, the mammary glands were evaluated for proliferation and morphology of T47D derivative breast cancer epithelial cells inside the mouse mammary gland (i.e. *ex vivo*). At the end of the experiments, longitudinal mammary gland sections were processed for H&E staining and immunohistochemistry. For identification of human specific breast cancer cells, the CK18 (M7010) biomarker was used, and Ki67 (8D5) was used as a marker to assess proliferation. To examine estrogen responsiveness, PR-B (B-30) and EGFR (Ab-10) expression was determined in the treated and untreated group. After injection of the T47D variant cell lines into the mouse mammary glands the number of proliferative lesions were counted. Specifically, the H&E slides for each gland were scanned using the 4x objective at different fields to cover the entire gland. The images were captured and the solid tumor-like patches were quantified. Four glands for each cell line were counted to obtain the number of proliferative lesions and statistical significance was determined using a one-way ANVOA nonparametric test. All procedures involving these animals were conducted in compliance with state and federal laws, standards of the U.S. Department of Health and Human Services, and guidelines established by the IIT Research Institute Animal Care and Use Committee. The facilities and laboratory animal program is accredited by the Association for the Assessment and Accreditation of Laboratory Animal Care.

### Immunofluorescent staining of tissue sections of BCa-Mouse mammary glands

Human breast invasive lobular carcinoma formalin fixed paraffin embedded block samples (Pantomics, Richmond, CA, USA) were processed according to the manufacturer's instruction. All other tissue sections were deparaffinized by treating slides with 100% Xylene twice for 10 min, then rehydrating the slide by incubating with various concentrations of ethanol (i.e. 95%, 70% and 30%) for 10 min each. All slides were rinsed with deionized distilled H_2_O for 5 min and processed for antigen retrieval by incubation with 0.01 M citrate buffer (pH 6.0) in a steamer for 40 min at >95°C. After unmasking the slides with citrate buffer, they were blocked with Mouse on Mouse (M.O.M.^®^) Blocking Reagent (Vector Laboratories, Burlingame, CA, USA) according to the manufacturer's instruction. The slides were then incubated for 2 h at room temperature or 4°C overnight with primary antibodies including: mouse anti-CK18 (M7010) (Dako North America, Inc., CA, USA), rabbit anti-Ki67 (8D5) (Neomarkers, Freemont, CA, USA), mouse anti-EGFR (Ab-10) (Neomarkers), mouse anti-aromatase and mouse anti-PR-B at a 1:100 dilution in protein diluent solution (1% BSA). The cells were washed with 1× PBS three times and incubated with secondary anti-mouse Alexa594 or secondary anti-rabbit Alexa488 (Invitrogen) for 30 min. Afterwards the samples were mounted with Vectashield^®^ Antifade Mounting Media containing DAPI for fluorescence (Vector Laboratories Inc.) and stored in the dark at 4°C. Immunofluorescent staining was examined using an Olympus BX 40 microscope (Olympus) fitted with filters to detect both Alexa 594 and Alexa488.

### Flow cytometry for PR-B expression in T47D derivative cell line

The T47D variant cells (100,000 cells/ml) were fixed in ice cold buffered formalin, permeabilized with ice-cold methanol, rinsed in PBS and then incubated for 1 h with anti-PR-B primary antibody (B-30) (1:400) followed by anti-mouse Alexa 594 labeled secondary antibody (1:800). The cells were then subjected to flow cytometry analysis (Beckman Coulter Legacy MoFlo). The data were analyzed by SummitV3.4 flow cytometry analysis program and represented as mean florescence intensity (MFI, an arbitrary unit) obtained from triplicates.

### Statistical analysis

Data were summarized as the mean±standard error of the mean (s.e.m.) using the Graph Pad Prism V.6 software program. Two-way analysis of variance models were employed to compare relative cell proliferation between test groups and control followed by Bonferroni post-test for individual experiments. Differences between means were considered statistically significant when the *P*-value was less than 0.05. Results are expressed as the mean unit ±s.e.m. (****P<0.0001, ***P<0.001, **P<0.01, *P<0.05).

## Supplementary Material

Supplementary information
