# Supplementary material for: A novel and cost-effective ex vivo orthotopic model for the study of human breast cancer in mouse mammary gland organ culture
Source: Biol Open. 2020 May 29;9(5):bio051649. doi: 10.1242/bio.051649 (PMC7272353; doi:10.1242/bio.051649)
Supplement: Supplementary information [file biolopen-9-051649-s1.pdf]

Supplemental figures

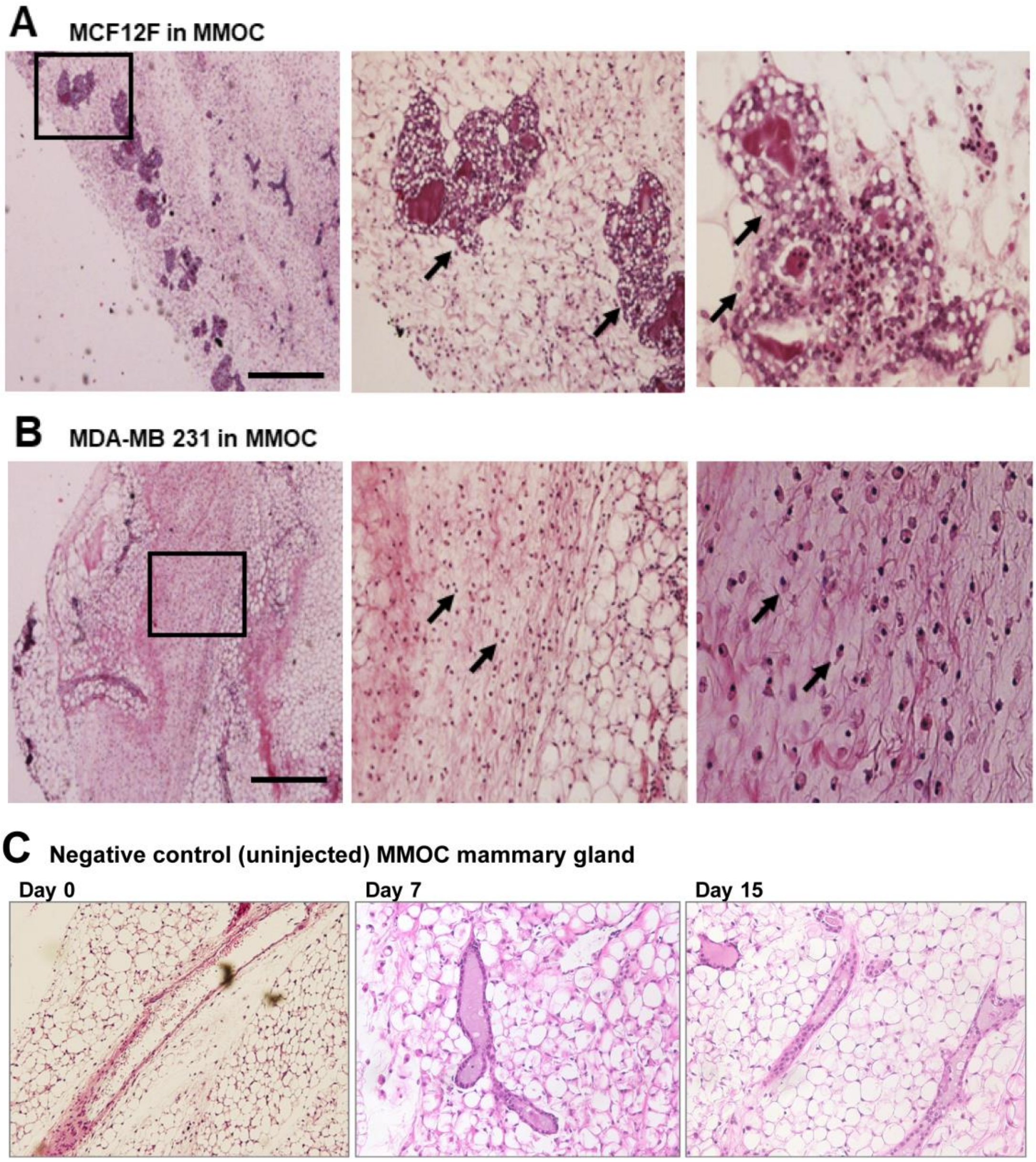

Figure S1. Establishment of the MCF-12F and MDA-MB 231 cells in the MMOC. Normal immortalized MCF-12F breast epithelial cells (top panel) and metastatic MDA-MB 231 triple negative cells (middle panel) were injected into the mouse mammary gland in *ex vivo* conditions and hematoxylin and eosin staining were conducted to examine growth patterns. The bottom panel represents mammary glands in the absence of injected cells.

Biology Open • Supplementary information

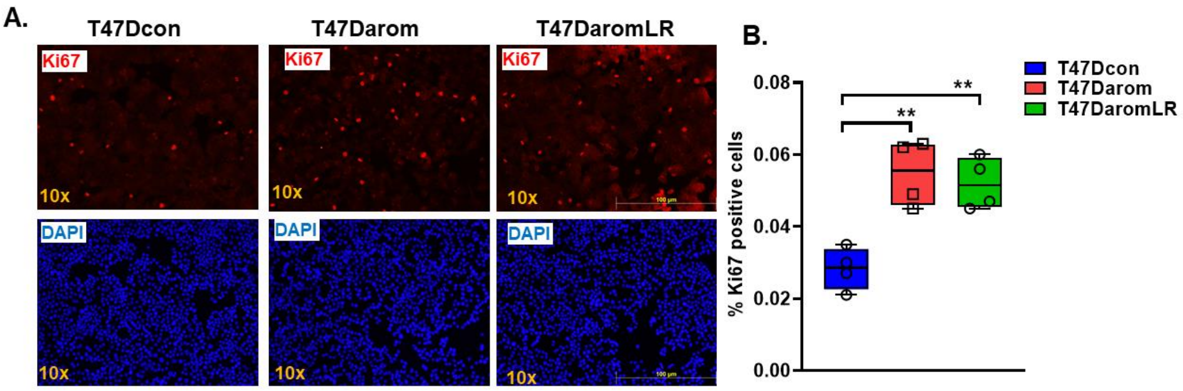

Figure S2. Aromatase expressing T47D cells exhibit a higher proliferative index. (A) T47Dcon, T47Darom and T47DaromLR cells were cultured *in vitro* and immunostained for Ki67 (in red) and DAPI (in blue). **(B)** The Ki67 positive cells were quantitated and graphed.

Biology Open • Supplementary information

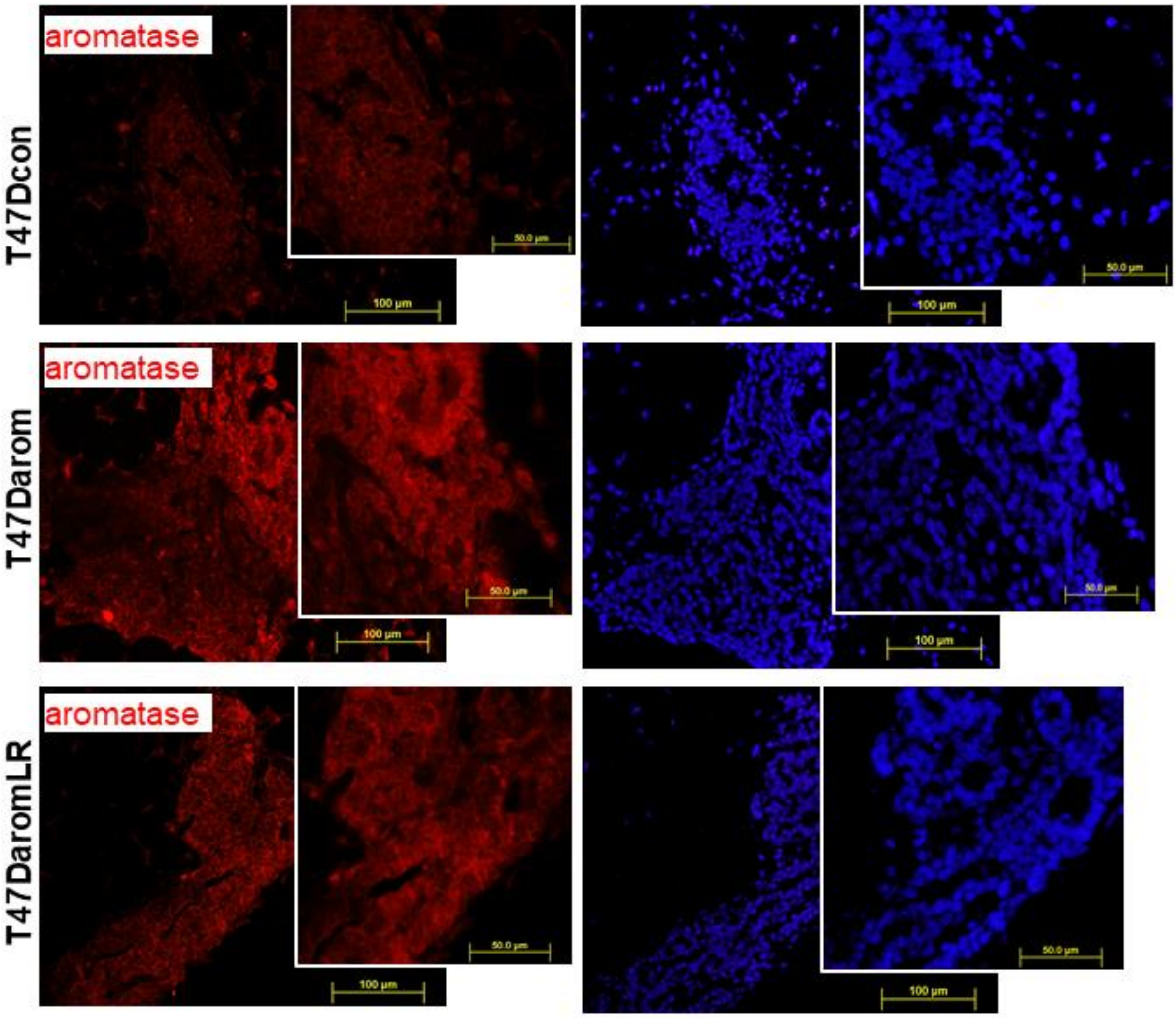

Figure S3. T47Darom and T47DaromLR cells express higher levels of aromatase in the BCa-MMOC model. T47Dcon, T47Darom and T47DaromLR cells were grown in the BCa-MMOC and mammary gland sections were immunostained for aromatase (in red) and DAPI (in blue). The inset area represents 40x images of the corresponding T47D variant cells in the BCa-MMOC.

Biology Open • Supplementary information

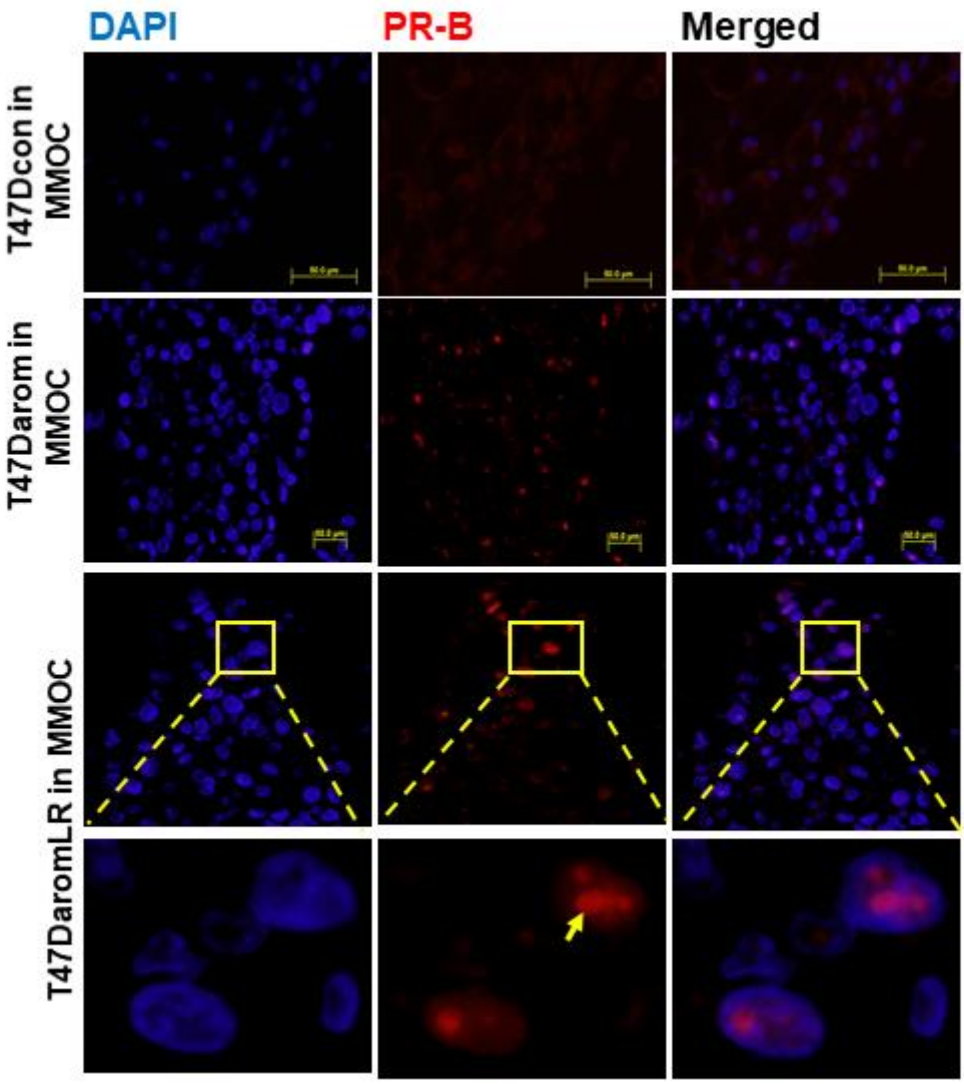

Figure S4. Immunofluorescence analysis of PR-B expression. T47Dcon, T47Darom and T47DaromLR were grown in the BCa-MMOC and mammary gland sections were immunostained for PR-B (in red) and DAPI (in blue). The bottom panel represents 40x images of the corresponding yellow boxed, inset areas of the T47DaromLR cells in the BCa-MMOC.

Biology Open • Supplementary information

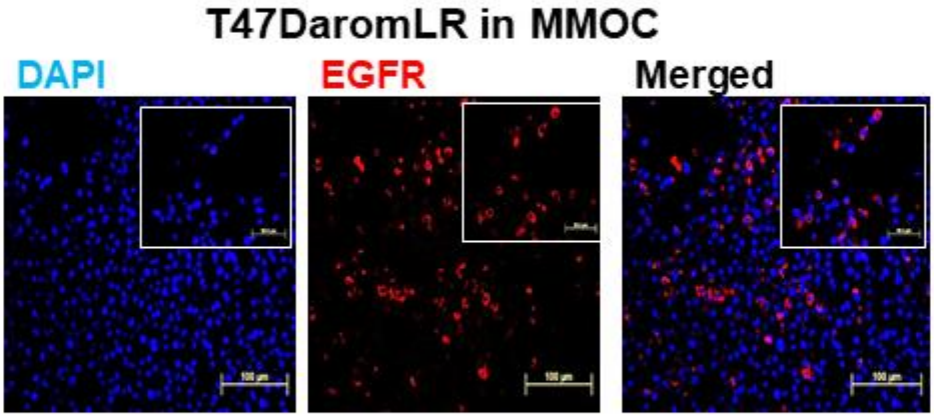

Figure S5. Immunofluorescence analysis of EGFR expression. T47DaromLR were grown in the BCa-MMOC and mammary gland sections were immunostained for EGFR (in red) and DAPI (in blue).

Biology Open • Supplementary information
